# Characterization and Expression Analysis of SOLD1, a Novel Member of the Retrotransposon-Derived Ly-6 Superfamily, in Bovine Placental Villi

**DOI:** 10.1371/journal.pone.0005814

**Published:** 2009-06-05

**Authors:** Koichi Ushizawa, Toru Takahashi, Misa Hosoe, Keiichiro Kizaki, Kazuyoshi Hashizume

**Affiliations:** 1 Reproductive Biology Research Unit, Division of Animal Sciences, National Institute of Agrobiological Sciences, Tsukuba, Ibaraki, Japan; 2 Department of Veterinary Medicine, Faculty of Agriculture, Iwate University, Morioka, Iwate, Japan; New Mexico State University, United States of America

## Abstract

**Background:**

Ly-6 superfamily members have a conserved Ly-6 domain that is defined by a distinct disulfide bonding pattern between eight or ten cysteine residues. These members are divided into membrane-type and secretory-type proteins. In the present study, we report the identification of a novel Ly-6 domain protein, secreted protein of Ly-6 domain 1 (SOLD1), from bovine placenta.

**Principal Findings:**

*SOLD1* mRNA was expressed in trophoblast mononucleate cells and the protein was secreted into and localized in the extracellular matrix of the mesenchyme in cotyledonary villi. SOLD1 bound mainly with type I collagen telopeptide. We confirmed secretion of SOLD1 from the basolateral surface of a bovine trophoblast cell line (BT-1). It may be related to the organization of the extra-cellular matrix in the mesenchyme of fetal villi. Since trophoblast mononucleate cells are epithelial cells, their polar organization is expected to have a crucial role in the SOLD1 secretion system. We established that *SOLD1* is an intronless bovine gene containing the *Alu* retrotransposon, which was integrated via cytoplasmic reverse transcription.

**Conclusion:**

We identified a novel retrotransposon-like Ly-6 domain protein in bovine placenta. SOLD1 is a crucial secreted protein that is involved in the organization of the mesenchyme of the cotyledonary villi. Furthermore, the gene encoding *SOLD1* has an interesting genomic structure.

## Introduction

Ruminants form the cotyledonary placenta at the feto-maternal interface. Two specific types of trophoblast cells, trophoblast giant binucleate cells (BNCs) and trophoblast mononucleate cells (TMCs), play a crucial role in ruminant placentation [1], [2]. The properties of BNC-specific genes, such as anti-apoptotic BCL2-related protein A1 (BCL2A1), which is involved in cell maintenance [3], placental lactogen (CSH1) [1], [4], [5], [6], prolactin-related proteins (PRPs) [7], and pregnancy-associated glycoproteins (PAGs) [8], have been investigated, and TMC-expressed interferon-tau (IFNT) is the molecule for maternal recognition of pregnancy. BNC and TMC individually produce numerous proteins of unknown function. It is important to identify the genes that are specifically expressed in each cell type in order to systematically decipher the function of the trophoblast cells.

In a recent gene expression profiling analysis using a bovine placental-specific microarray, we detected the specific expression of a novel gene during the peri-implantation period [9]. This bovine gene is composed of only one Ly-6 (lymphocyte antigen-6, Ly-6/urokinase-type plasminogen activator receptor, uPAR) domain and a signal peptide. We named this gene “secreted protein of Ly-6 domain 1”, and assigned it a gene symbol of “*SOLD1*”. Normally, the Ly-6 domain consists of 70–100 amino acids and is characterized by a conserved pattern of 8 or 10 cysteine residues that have a defined pattern of disulfide bonding in several types of proteins [10], [11], [12]. The Ly-6 superfamily is divided into two groups, membrane-type GPI-anchored proteins and secretory proteins. Most members of this family are membrane-type proteins, such as the complement regulatory protein CD59 [13], [14], [15], plasminogen activator, urokinase receptor (PLAUR, uPAR) [10], [12], [16], lymphocyte antigen 6 complex, and locus D (LY6D, E48) [17], [18], [19]. The secreted members of this family, such as secreted LY6/PLAUR domain containing 1 (SLURP1) [20], [21], Ly6/neurotoxin 1 (LYNX1, SLURP2) [22], [23], [24], acrosomal vesicle protein 1 (ACRV1, SP10) [25], [26], [27], expressed in prostate and testis (PATE) [28], [29], [30], and secreted seminal vesicle Ly6 protein (Sslp-1) [31], lack the GPI-anchor. To date, no evidence has been reported that the members of the Ly-6 superfamily have a common function. For example, CD59 is an inhibitor of the complement cascade and regulates immunosuppression [14], [15]. PLAUR has a crucial role in proteolysis of extracellular matrix proteins [32], [33]. SLURP1 and LYNX1 control the pro- and anti-apoptosis, respectively, of the keratinocyte through the nicotinic cholinergic receptor (nAChR) [23], [34]. ACRV1, PATE, and Sslp-1 are sperm-associated proteins with a possible role in mammalian sperm maturation. Each Ly-6 protein is reported to have a different function. Preliminary evidence suggests that SOLD1 also has a specific function in the placenta [25], [30], [31].

In the present study, we identified another specific feature of SOLD1. Secreted SOLD1 protein was detected under the basement membrane, but only trophoblasts expressed the *SOLD1* gene. There is some evidence that trophoblast cells have bilateral secretion ability [35], [36], [37]. Some trophoblast cells have the same polarity as epithelial cells, and are able to release some enzymes and cytokines at both the apical and/or the basolateral surface. For example, the bilateral secretion of interferon-gamma has been confirmed in a porcine trophoblast cell line [35]. In contrast, the basolateral secretion of matrix metalloproteinase-2 and -9 (MMP2 and MMP9) has been confirmed in human syncytiotrophoblasts [36]. The secretion of leptin was confirmed at both the apical and basolateral surfaces of the human trophoblast cell line BeWo [37]. The cotyledonary villi are composed of the trophoblast and mesenchyme. We explored the possibility that SOLD1 has some function in the mesenchyme when it is secreted in the direction of the basement membrane. The mesenchyme is the connective tissue that contains much extra-cellular matrix (ECM). The binding properties of ECM and SOLD1 were important clues in our search for the function SOLD1.

Here, we studied SOLD1, a novel and crucial TMC-secreted protein, and examined its secretion polarity from TMC, along with the temporo-spatial expression of *SOLD1*. We also investigated SOLD1 binding properties to the ECM. Since the *SOLD1* gene contains a retrotransposon in the bovine genome, we further explored the genomic properties of this gene.

## Results

### mRNA expression of SOLD1

**Figure 1:**
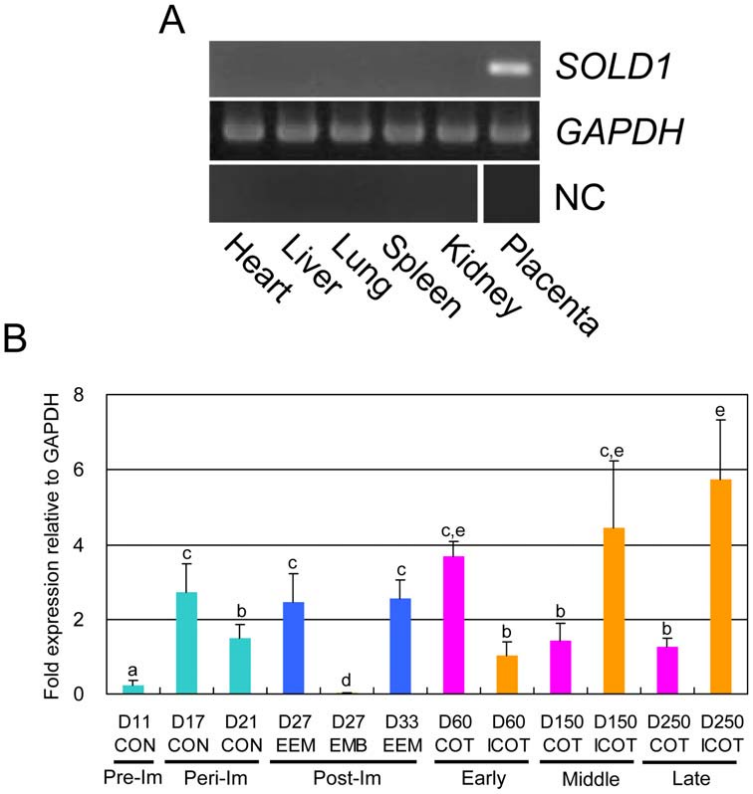
Expression of *SOLD1* mRNA. (A) Expression of *SOLD1* mRNA in various bovine tissues, including heart, liver, lung, spleen, and kidney, was analyzed by RT-PCR. Cotyledonary tissue at Day 150 of gestation was used as a bovine placental sample. *GAPDH* expression in each tissue is presented as a positive control.*CSH1* expression in each tissue except for the placenta is presented as a negative control (NC). *ALB* expression in the other tissue is presented as a negative control (NC).(B)Quantitative expression of SOLD1 in bovine placental tissues during the initial to rate stage of pregnancy by qRT-PCR analysis. Pre-Im, pre-implantaion; Peri-Im, peri-implantation; and Post-Im, post-implantation. CON, conceptus; EEM, extra-embryonic membrane; EMB, embryo; COT, cotyledon; and ICOT, intercotyledon. D, day after insemination. Expression of these mRNAs was normalized to the expression of *GAPDH* measured in the corresponding RNA preparation. Values are means±SEM. Values with different letters (a, b, c and d) are significantly different (P<0.05).

Figure 1A depicts the tissue distribution of *SOLD1*, as determined by RT-PCR analysis. No *SOLD1* mRNA expression was detected in the heart, liver, lung, spleen, and kidney tissues. In contrast, *SOLD1* mRNA was found in the placenta (cotyledon).

**Figure 2:**
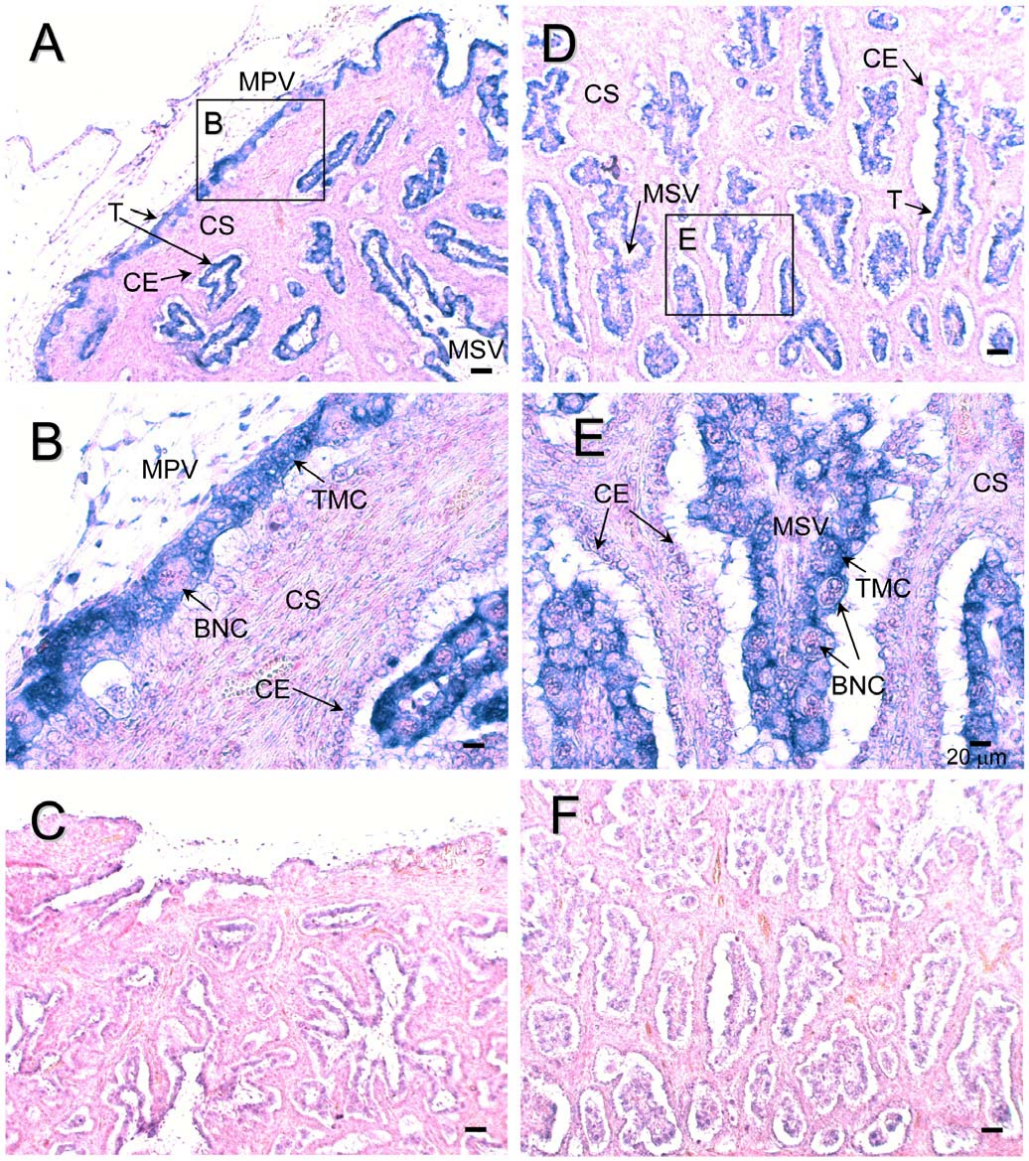
mRNA localization of *SOLD1* in the bovine placentome on Day 60 of gestation. *SOLD1* mRNA was detected by in situ hybridization. (A, D) DIG-labeled anti-sense cRNA probes were used. (B, E) Enlarged images of frames in A and D, respectively. (C, F) DIG-labeled sense cRNA probes were used. CE, caruncular epithelium; CS, caruncular stroma; T, trophoblast; TMC, trophoblast mononucleate cells; BNC, trophoblast binucleate cells; MPV, mesenchyme of primary villi; and MSV, mesenchyme of secondary villi. Scale bars = 100 µm (A, C, D and F) and 20 µm (B and E).

Quantitative expression of *SOLD1* is depicted in Fig. 1B. In ovoid-shaped conceptus on Day 11, expression of *SOLD1* was stable, but barely detectable. In the extra-embryonic membrane (EEM) on Day 17 to 34, expression of *SOLD1* was consistently detected, but the expression level was temporarily reduced on Day 21 (Fig. 1B). In the cotyledon (COT: villous trophoblast), the expression of *SOLD1* decreased after Day 60 of gestation. In contrast, the expression increased after Day 60 of gestation in the intercotyledon (ICOT: extravillous trophoblast, the areas between cotyledonary villi) (Fig. 2A, B). We determined the localization of *SOLD1* mRNA by in situ hybridization on Day 60 of bovine gestation (Fig. 2). *SOLD1* was expressed in TMCs in the COT and the ICOT. Small amounts of *SOLD1* mRNA were detected in the maternal tissues (caruncle and intercaruncle endometrium). No significant signal was detected with the sense probes.

### Localization of SOLD1 protein

**Figure 3:**
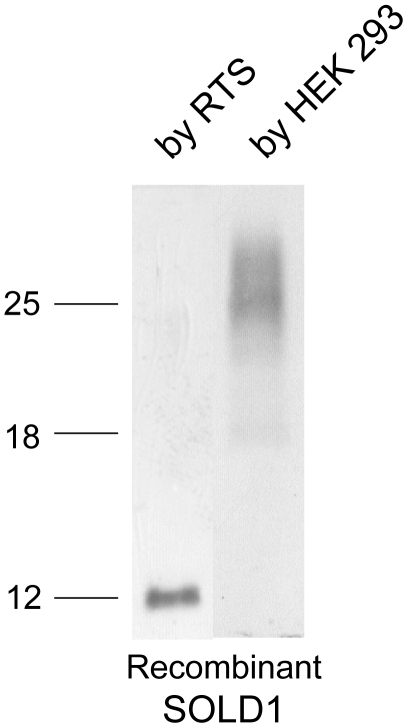
Western blot analysis of recombinant SOLD1 proteins. Conditioned media from HEK 293 cells transiently transfected with the bovine *SOLD1* gene were collected. The protein was also produced by RTS. The purified proteins (1 ng) were loaded onto separate lanes. The proteins were separated by SDS-PAGE and specific proteins were detected by western blot analysis using a bovine anti-SOLD1 antibody.

First, we examined the specificity of the custom-made anti-bovine SOLD1 (anti-bSOLD1) antibody. Recombinant SOLD1 proteins were made using either HEK 293 cells or Rapid translation systems (RTS; Roche Diagnostics, Basel, Switzerland). The SOLD1 protein produced by HEK 293 cells was approximately 25 kDa, and included sugar chains, as detected by western blot analysis (Fig. 3). The SOLD1 protein synthesized by RTS was approximately 12 kDa, and lacked sugar chains (Fig. 3).

**Figure 4:**
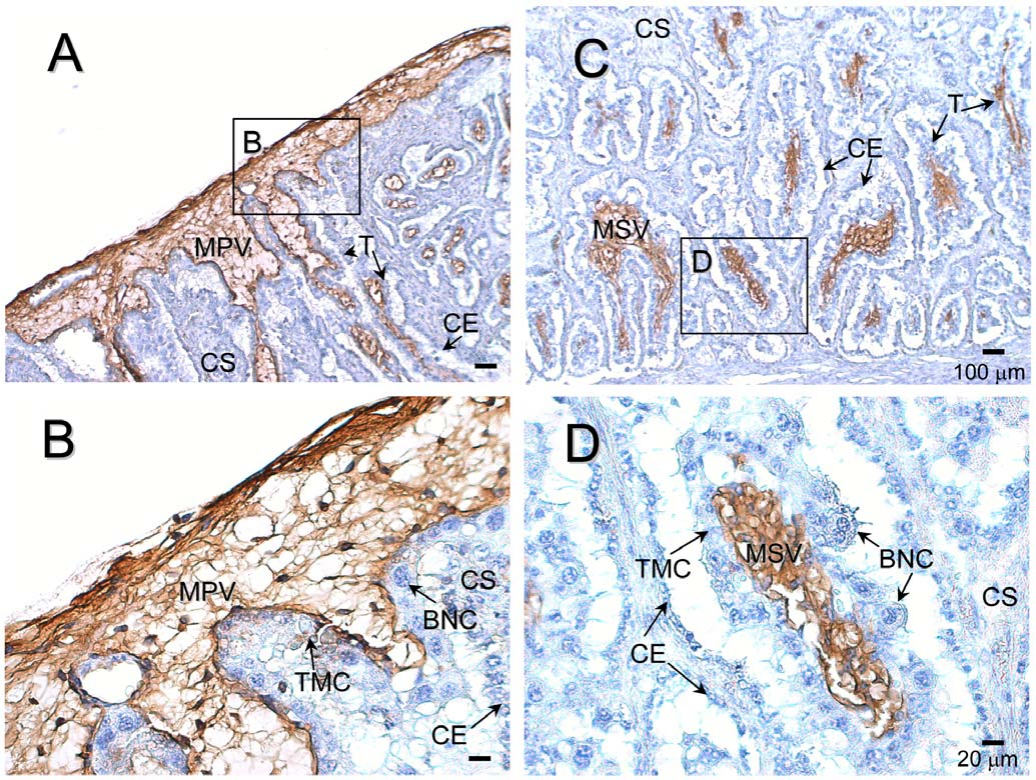
Protein localization of SOLD1 in the bovine placentome on Day 60 of gestation. (A, C) SOLD1 protein was detected by immunohistochemistry. (B, D) Enlarged images of frames in A and C, respectively. Custom-made bovine anti-SOLD1 antibody was used. CE, carunclar epithelium; CS, carunclar stroma; T, trophoblast; TMC, trophoblast mononucleate cells; BNC, trophoblast binucleate cells; MPV, mesenchyme of primary villi; and MSV, mesenchyme of secondary villi. Scale bars = 100 µm (A and C) and 20 µm (B and D).

The results of immunohistochemistry using the anti-bSOLD1 antibody are shown in Fig. 4. Intense staining of the SOLD1 protein was detected in the mesenchyme area of stem (primary) and branch (secondary) villi in the COT. Weak staining was found in TMC, which expressed *SOLD1* mRNA (as seen in Fig. 2). The protein signal was absent in both the carucular and intercaruncular regions of the maternal tissues (Fig. 4).

### SOLD1 binding properties to ECM

**Figure 5:**
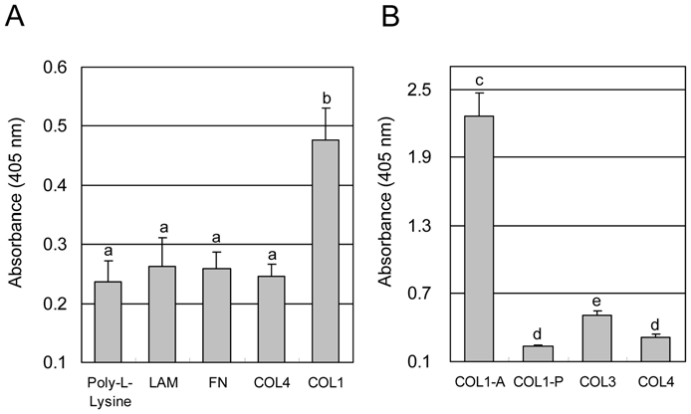
Recombinant SOLD1 binding assay to ECM-coated plate. (A) Absorbance of SOLD1 binding to 96-well microtiter plates coated commercially with ECM components type I collagen (COL1), type IV collagen (COL4), fibronectin (FN), and laminin (LAM), and the negative control, poly-L-lysine (PLL). (B) Absorbance of SOLD1 binding to 96-well microtiter plates coated manually with ECM components (300 µg/well each) telopeptide including type I collagen (tropocollagen, COL1-A), telopeptide excluding type I collagen (atelocollagen, COL1-P), type III collagen (COL3), and type IV collagen (COL4). The means±SEM of triplicate measurements are shown. Values with different letters are significantly different (P<0.05).

Specific binding was detected between SOLD1 and the type I collagen (COL1) coated plate (Fig. 5A). No specific binding was detected between SOLD1 and any other ECM. SOLD1 bound specifically to type I collagen (COL1-A) telopeptide, and weakly to type III collagen (COL3) (Fig. 5B).

### Localization of type I and type III collagens

**Figure 6:**
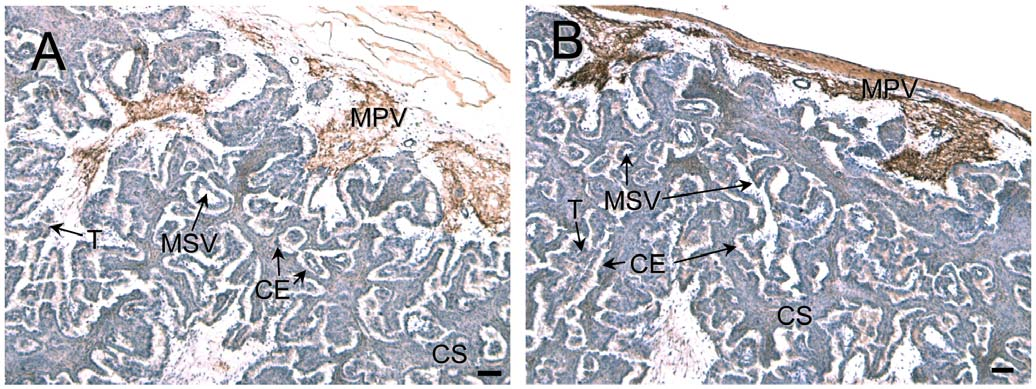
Protein localization of COL1 and COL3 in the bovine placentome on Day 60 of gestation. (A) COL1 protein was detected in the bovine placentome. (B) COL3 protein was detected in the bovine placentome. CE, carunclar epithelium; CS, carunclar stroma; T, trophoblast; MPV, mesenchyme of primary villi; and MSV, mesenchyme of secondary villi. Scale bars = 100 µm.

COL1 was detected in the caruncular epithelium, caruncular stroma, trophoblast, mesenchyme of primary villi, and the interstice area between COT and the caruncle, and faint staining was detected in the mesenchyme of secondary villi (Fig. 6A). Similar expression patterns were found using the COL3 antibody (Fig. 6B).

### Apico-basal polarity of SOLD1 secretion from BT-1 cells

**Figure 7:**
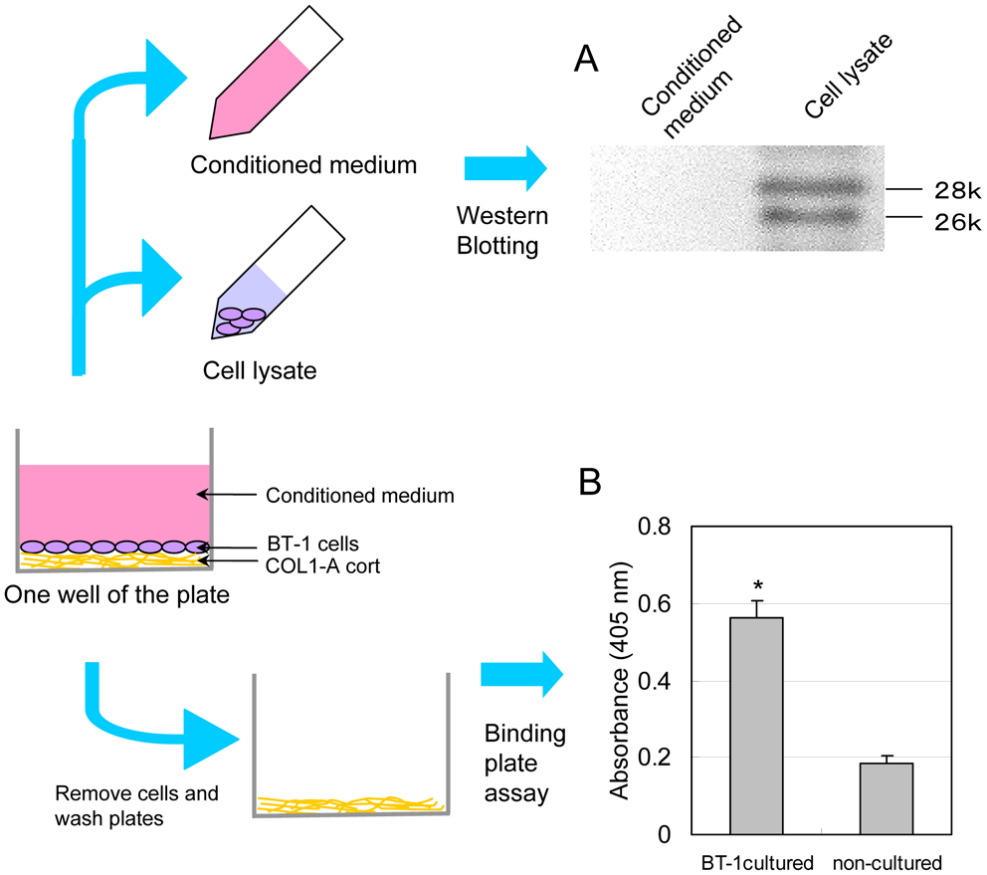
Apico-basolateral polarity of SOLD1 secretion from BT-1 cells. (A) Western blot analysis of BT-1 conditioned medium and BT-1 cell lysate. (B) Results of the SOLD1 binding assay. BT-1 cells were cultured and removed on COL1-A coated plates. The plates were washed ten times with TBST. SOLD1 binding to COL1-A coated plates was measured by monitoring absorbance at 405 nm in each of four wells per sample. The means±SEM of triplicate measurements are shown. Values with asterisks are significantly different (P<0.05).

From the gene expression data in Fig. 2 and the protein expression data in Fig. 4, we hypothesized that TMCs secreted SOLD1 at their basolateral surfaces. We examined the in vitro secretion of TMCs using the bovine trophoblast cell line, BT-1. Western blot analysis did not detect any SOLD1 protein in BT-1 cell conditioned medium (Fig. 7). In contrast, 28-kDa SOLD1 protein was detected in the BT-1 cell lysate (Fig. 7). The protein was detected on the collagen-coated plate after the cultured BT-1 cells were removed from the binding assay plate (Fig. 7). We confirmed that the protein was secreted to the basolateral surface of the trophoblast (BT-1) cell.

### Properties of mRNA and deduced amino acid sequences

**Figure 8:**
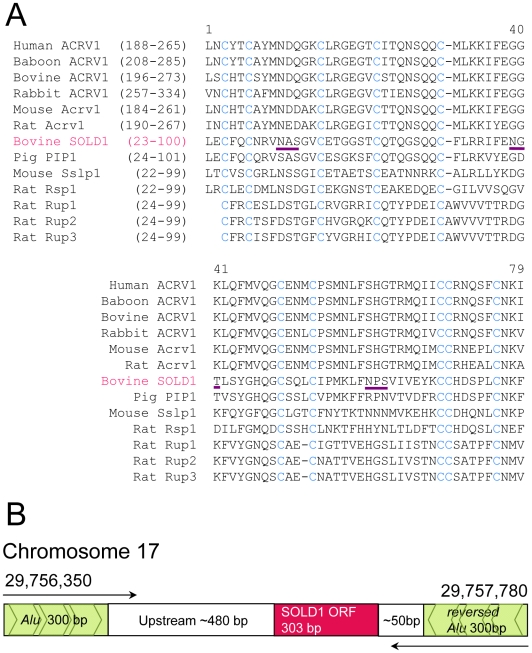
Comparison of the Ly-6 domain sequence and the genome structure of *SOLD1*. (A) Comparison of amino acid sequences between ruminant SOLD1 and the phylogenetically neighbouring Ly-6 domains in each protein selected by ProDom. Residues identical in all Ly-6 domains are shown by blue letters. Sequence gaps are shown by hyphens.The potential *N*-glycosylation site is underlined in purple.(B) The genome structure of the *SOLD1* region (genome sequence position 29,756,350 to 29,757,780) in bovine chromosome 17, as determined by NCBI MapView.

We cloned a full-length cDNA of *SOLD1* from the bovine placentome and identified the 303-bp open-reading-frame (ORF) cDNA. The amino acid sequence deduced from full-length *SOLD1* cDNA was 100 amino acids (aa). The *N*-terminal region (22-aa) of the SOLD1 protein was rich in hydrophobic amino acid residues, which are characteristic of a signal peptide (Fig. 8A). There was 31∼73% similarity between SOLD1 and rat urinary proteins (Rup-1, Rup-2, Rup-3), rat spleen protein (Rsp-1), pig protein (PIP-1), and mouse Sslp-1, and the C-terminal regions of ACRV1 proteins from various species, when domain retrieval was carried out based on ProDom [http://prodom.prabi.fr/prodom/current/html/home.php] (Fig. 8A and Table 1). Although identity among the amino acid sequences was low, the Cysteine (Cys) configuration was completely conserved in all proteins. This conserved region is called the Ly-6 domain [22], [23]. The mature SOLD1 protein was predicted to consist of only one Ly-6 domain. SOLD1 had three consensus sequences for *N*-glycosylation (Asn-X-Ser/Thr) at amino acid positions 32 to 34, 60 to 62, and 81 to 83 (Fig. 8A). We submitted the mRNA sequences to the DNA Data Bank of Japan (DDBJ). The DDBJ/GenBank accession number is AB297495.

**Table 1:** Similarity between bovine SOLD1 and the Ly-6 domain of related molecules.

Protein name	Similarity for SOLD1 (%)
Pig PIP1	73
Mouse Sslp1	38
Rat RSP1	33
Rat RUP1	32
Rat RUP2	32
Rat RUP3	32
Human ACRV1	42
Baboon ACRV1	42
Bovine ACRV1	41
Rabbit ACRV1	38
Mouse ACRV1	42
Rat ACRV1	38

### Genome organization of SOLD1

A high level of similarity between bovine *SOLD* equences was identified on chromosomes 17 (chr17) and 29 (chr29) using the NCBI BLAST search. On chr17, the full-length *SOLD1* ORF was located between nucleotides 29,757,120 and 29,757,423, and lacked an intron. We established that the *SOLD1* ORF on chr17 is flanked by reversed *Alu* sequences (*Alu* - upstream region - ORF - reversed *Alu*) (Fig. 8B). This feature was similar to the structure of the *Alu* integrated retrotransposon. On chr29, nucleotides 1-64 of the *SOLD1* ORF were located between nucleotides 24,532,076 and 24,532,139, and nucleotides 179–303 were located between nucleotides 24,537,293 and 29,537,417. However, the sequence of the portion between nucleotides 65–178 on chr17 was missing on chr29.

## Discussion

In cattle, *SOLD1* was mainly expressed in fetal tissues, such as the conceptus, placentome, and ICOT, throughout gestation (Fig. 1B). SOLD1 may have the specific role of building up the fetal placentomal architecture. As shown in Fig. 2 and 4, TMCs produced SOLD1, and secreted it into the mesenchyme.

COL1 was a component of connective tissue in the placentome, and supported the mesenchyme of primary villi and the trophoblast (Fig. 6) [38], [39]. We established that SOLD1 specifically bound to the telopeptide in fibrillar type COL1, because it bound to the tropocollagen (COL1-A) but not to the atelocollagen (COL1-P) (Fig. 5). Moreover, SOLD1 also bound slightly to the reticular type COL3 (Fig. 5). Various kinds of growth factors, cytokines, and hormones, such as platelet-derived growth factor (PDGF) [40], [41], hepatocyte growth factor (HGF) [42], transforming growth factor beta 1 (TGF-beta1) [43], [44], tumor necrosis factor alpha (TNF-alpha) [45], interleukin-2 (IL2) [46], interleukin-7 (IL7) [47], oncostation M (OSM) [48], and prolactin-related protein-I (PRP1) [49], bind ECM, and they may perform a pivotal role in the modulation of local bioavailability. SOLD1 may also participate in ECM remodeling.

The bovine placentomal trophoblast cells consist of two distinct cell types, BNCs and TMCs [50]. BNCs account for approximately 20% of the total trophoblast cell population [50]. BNCs are directly involved in modifying the uterine epithelium, beginning at implantation and continuing until term. [1], [50], [51] Hence, BNCs plays a pivotal role in fetomaternal communication in cattle. BNCs possess two nuclei and large populations of characteristic granules that produce an array of compounds [2]. BNCs secrete placental-specific prolactin-related hormones (placental lactogen, prolactin-related proteins), matrix protease-related molecules (i.e., heparanase, MMP14, TIMP2), aspartic protease family (i.e., pregnancy-associated glycoproteins), steroid hormones, and prostanoids [2], [5], [6], [7], [8], [52], [53]. In the current study, we provide evidence that TMCs are the primary cells responsible for SOLD1 production. TMCs have been known to produce interferon-tau (IFNT), which is a substance for maternal recognition of pregnancy in ruminants. This cytokine is specifically produced in TMCs during the pre-implantation period [54], [55], [56]. IFNT acts on the uterine epithelium and maintains corpus luteum function [57]. The actual functions of SOLD1 are not currently clear; however, it may have a role in ECM degradation, as do the cathepsins (CTSs) and the trophoblast Kunitz domain proteins (TKDPs). CTSs are proteases that have biological roles in degrading ECM, catabolizing intracellular proteins, and processing pro-hormones. CTSs were expressed in both bovine BNCs and TMCs [58]. TKDPs are trophoblast-specific serine protease inhibitors in ruminants [59], [60]. The localization of TKDPs has not been established; however, several TKDPs were expressed either in BNCs or in TMCs, or in both BNCs and TMCs. SOLD1 was only expressed in TMCs, and not in BNCs. SOLD1 thus has the potential to be used as a marker gene of TMCs.

SOLD1 was secreted at the basolateral surface of TMCs (Fig. 7). ECM-degrading metalloproteinases, such as MMP2 and MMP9, are known to be basolateral secreting molecules in the human syncytiotrophoblast [36]. These results suggest that SOLD1 participates in the remodeling of ECM and the proliferation of fetal villi.

Sequences similar to SOLD1 were detected in other species by BLAST analysis (Fig. 8A). Although the percentage of similarity was not high between these proteins, the Cys configuration was completely conserved in each encoded protein. All of the proteins examined had at least one potential *N*-glycosylation site. We anticipate that these molecules evolved from a single origin. However, we do not yet know whether these genes have a common function, because the gene is detectable in various tissues.

The *SOLD1* mRNA sequence, as determined by genomic BLAST analysis, was retrieved on chr17 and chr29. The *SOLD1* sequence on chr17 contained a retrotransposon-like structure, flanked by forward and reverse *Alu* sequences (Fig. 8B). Two partial mRNA fragments of the *SOLD1* sequence coincided with the sequence on chr29. The bovine *ACRV1* gene is located in the vicinity of the above *SOLD1* partial fragments on chr29 (nucleotide 24,507,600 to 24,514,200). The Cys configuration of the Ly-6 domain in SOLD1 and bovine ACRV1 are completely identical. ACRV1 specifically appears in the spermatozoon [25], [26], [27], and it has been suggested that this protein has a function related to fertility potential [61]. We predict that *SOLD1* and *ACRV1* evolved from the same origin on chr29, and that both have a reproductive function. Therefore, *SOLD1* might have been transferred from chr29 to chr17 in some cases. We suggest that *SOLD1* is a retrotransposon transferred from the bovine cytoplasmic genome via mRNA. No Ly-6 domain protein has thus far been reported for the intronless sequences of primates or rodents. This genome structure would be an interesting difference between species if it were specific for cattle. Placental mammals may have evolved by the insertion of retrotransposons [62], and the insertion of *SOLD1* may have given rise to ruminants.

The Ly-6 superfamily has been detected in various tissues. ACRV1 structurally resembles SOLD1, and is a spermatid-specific gene in several species [25], [26], [63]. Mouse Sslp-1 is also a spermatid-specific gene [31]. Rat Rup-1, Rup-2, and Rup-3 are expressed in urinary organs, and rat Rsp-1 is expressed in the spleen [10]. SOLD1 was mainly expressed in placental tissues (Figs. 1 and 2). Recently, the expression of PATE-P and –Q (Pate-P and –Q) was demonstrated in human and mouse placental tissue [29]. Mouse Pate-P was reported to modulate the activity of the alpha4beta2 heteromeric nicotinic acetylcholine receptor (nAChR). SOLD1 may have a similar role as PATE-P in the placenta.

In conclusion, we identified the secreted protein SOLD1, which contains the Ly-6 domain. *SOLD1* mRNA appeared in TMCs in the bovine placenta, and its protein localized in the mesenchyme of primary and secondary villi. SOLD1 bound to the telopeptide of fibrillar type I collagen and the reticular type III collagen in mesenchyme villi. SOLD1 has basolateral secretion polarity in TMC. SOLD1 is related to the mesenchyme organization in villi. *SOLD1* was found to be an intronless structure in the bovine genome, and we deduced that the *Alu* sequence integrated as a retrotransposon of the cytoplasmic genome derivation through mRNA. We propose that retrotransposable SOLD1 organizes bovine cotyredonary villi.

## Materials and Methods

### Animal and tissue collection

All procedures for following animal experiments were carried out in accordance with the guidelines and ethics approved by the Animal Ethics Committee of the National Institute of Agrobiological Sciences for the use of animals. Bovine placental tissues for cDNA cloning, mRNA quantitative expression, in situ hybridization and immunohistochemistry were collected from Japanese Black cows. The conceptuses, extra-embryonic tissues and placenta were collected on days 11 (ovoid; n = 3 animals) after artificial insemination (designated as day 1 of pregnancy), 17 to 18 (pre-implantation; n = 3 animals), 20 to 21 (peri-implantation; n = 3 animals), 27 to 28 (post-implantation; n = 3 animals), 31 to 34 (initial; n = 3 animals), 56 to 64 (early; n = 4 animals), 144 to 149 (middle; n = 4 animals), 245 to 252 (late; n = 4 animals). After day 56, extra-embryonic tissues were separated into two portions, COT and ICOT. The EEM on days 27 to 28 and 31 to 34 contained fetal membrane with a few villi, as COT isolation from the membrane was difficult. It was also difficult to separate the trophoblastic and embryonic portions in the conceptuses (CON) on days 11, 17 to 18, and 20 to 21. Days 11, 17 to 18, 20 to 21, 27 to 28, 31 to 34, 56 to 64, 144 to 149, and 245 to 252 were named Day 11, Day 17, Day 21, Day 28, Day 32, Day 60, Day 150, and Day 250, respectively. The collected tissue samples were stored at −80°C until RNA extraction. The Day 60 placentomes were fixed in 3.7% formaldehyde PBS at pH 7.4 and then embedded in paraffin wax and stored at 4°C until in situ hybridization and immunohistochemical analysis.

### Cloning of full-length *SOLD1* cDNA

Full-length cDNA of bovine *SOLD1* was amplified from bovine cotyledonary tissue by RT-PCR. In brief, RNA was isolated from cattle placentomes using ISOGEN (Nippon Gene, Toyama, Japan). Genomic DNA was removed by DNase, using the Turbo DNA Free Kit (Ambion, Austin, TX, USA). The total RNA in a reaction mixture was subjected to reverse transcription and template cDNA synthesis using oligo(dT) primers and Superscript III Reverse Transcriptase (Invitrogen, Carlsbad, CA, USA) at 50°C for 50 min. PCR was performed with *SOLD1*-specific primers (Table 2). The *SOLD1* primers were designed from the LOC100125878 hypothetical bovine protein sequence (GenBank reference accession number NM_001105478). The products were cloned into a pGEM-T Easy Vector (Promega, Madison, WI, USA), and sequenced using an ABI Prism 370 automatic sequencer (Applied Biosystems, Foster City, CA, USA).

**Table 2:** Oligonucleotide primers used for cDNA cloning or RT-PCR analysis.

Gene	Primer	Sequence	Position*
*SOLD1*	Forward	5′ TCCAGAGATGGCTAAGTGCCTT 3′	50–71
(NM_001105478)	Reverse	5′ GAGTTGGACATGACTGAGCCAC 3′	453–432
*GAPDH*	Forward	5′ CCTTCATTGACCTTCACTACATGGTCTA 3′	71–98
(U85042)	Reverse	5′ GCTGTAGCCAAATTCATTGTCGTACCA 3′	927–901
*CSH1*	Forward	5′ AGAAGAACGAGCCCTATCCAGT 3′	642–663
(NM_181007)	Reverse	5′ TTTTGACATCTCTACAGAATCT 3′	960–939
*ALB*	Forward	5′ CTGAGCTTGATCCTGAACCGGTT 3′	1458–1480
(NM_180992)	Reverse	5′ TCTCAGTATCGGGAAGTGTGCAT 3′	1662–1640

* Position: The nucleotide position in each accession number.

### RT-PCR

The tissue distribution of *SOLD1* expression was examined by RT-PCR. *GAPDH* was used as a positive control. Since *SOLD1* is an intronless sequence, exon regions from placental lactogen (*CSH1*) and albumin (*ALB*) genes were used as a negative control to detect genomic contamination. Exon 5 of *CSH1* was used as a negative control, except when analyzing placental samples. Exon 12 of *ALB* was used as a negative control when analyzing the other tissues. Details of the RT-PCR method were described previously [3]. The total RNA in a reaction mixture was used for reverse transcription and template cDNA synthesis using oligo(dT) primers and Superscript III Reverse Transcriptase (Invitrogen) at 50°C for 50 min. Each PCR contained the cDNA template, primers, autoclaved milliQ water, and AmpliTaq Gold PCR Master Mix (Applied Biosystems). Denaturation took place at 95°C for 30 s and extension at 72°C for 1 min. Twenty-six cycles were performed for all samples. The annealing temperature was set at 57°C for 30 s. A single denaturation step at 95°C for 10 min before the first PCR cycle, and a final extension step at 72°C for 10 min after the last PCR cycle were also performed. The PCR products were analyzed by agarose gel electrophoresis and visualized by ethidium bromide staining. The same *SOLD1*-specific primers were used in the RT-PCR as in cDNA cloning (Table 2). The primers were commercially synthesized (Tsukuba Oligo Service, Tsukuba, Japan).

### Quantitative real-time RT-PCR (qRT-PCR)

Expression of *SOLD1* was quantitatively confirmed at each stage of gestation by qRT-PCR using the Power SYBR Green PCR Master Mix (Applied Biosystems). Fifty nanograms of total RNA were reverse-transcribed into cDNA for 30 min at 48°C using MultiScribeTM Reverse Transcriptase with a random primer, dNTP mixture, MgCl_2_, and RNase inhibitor. After heat inactivation of the reverse transcriptase for 5 min at 95°C, PCR and the resulting relative increase in reporter fluorescent dye emission were monitored in real time using an Mx3000P QPCR System (Stratagene, La Jolla, CA, USA). In the SYBR Green assay, primer pairs were designed using Primer Express Software (Applied Biosystems). The primers used to amplify each gene are listed in Table 3. Thermal-cycling conditions included an initial sample incubation at 50°C for 2 min and at 95°C for 10 min, followed by 40 cycles at 95°C for 15 s and at 60°C for 1 min. The relative differences in the initial amounts of each cDNA species were determined by comparing their threshold cycle (C_T_) values. To quantify the mRNA concentrations, standard curves for each gene were generated by serial dilution of the plasmid containing the corresponding cDNA. The dissociation curve for detecting the SYBR Green-based objective amplicon was confirmed, because SYBR Green also detects any double-stranded DNA, including primer dimers, contaminating DNA, and PCR products from misannealed primers. Contaminating DNA or primer dimers would show up as a peak separate from the desired amplicon peak. The expression ratio of each gene to *GAPDH* mRNA was calculated to adjust for variations in the RT-PCR reaction. All values are presented as means±SEM. The replication of qRT-PCR data was performed in biological replicates from n = 3 or 4 animals and n = 2 technical replicates per animal sample (for a total of six or eight data points). Statistical analysis was performed using one-way ANOVA followed by the Tukey-Kramer multiple-comparison test. Differences were considered significant at P<0.05.

**Table 3:** Oligonucleotide primers used for qRT-PCR analysis.

Gene	Primer	Sequence	Position*
*SOLD1*	Forward	5′ GGAAGCACCTGCCAGACTCA 3′	177–196
(NM_001105478)	Reverse	5′ AAAGCGTGCCATTTTCGAAG 3′	246–227
*GAPDH*	Forward	5′ AAGGCCATCACCATCTTCCA 3′	178–197
(U85042)	Reverse	5′ CCACTACATACTCAGCACCAGCAT 3′	253–230

* Position: The nucleotide position in each accession number

### *In situ* hybridization

The full-length *SOLD1* cDNA was used as template for hybridization probe synthesis. Digoxigenin (DIG)-labeled antisense and sense-complementary RNA probes were prepared as described in previous studies [3]. The placentomes were sectioned into 7 µm-thick sections. In situ hybridization was performed using the automated Ventana HX System Discovery with a RiboMapKit and BlueMapKit (Ventana, Tucson, AZ, USA). Briefly, bovine sections were hybridized with DIG-labeled probes in RiboHybe (Ventana) hybridization solution at 65°C for 6 h. The sections were washed three times in RiboWash (Ventana) (65°C; 6 min) after hybridization, and fixed in RiboFix (Ventana) (37°C; 10 min). The hybridization signals were then detected using a monoclonal-anti-digoxin biotin conjugate (Sigma, Saint Louis, MI, USA). Counter stain was performed using nuclear fast red (Ventana). After preparation, the hybridized slides were observed with a Leica DMRE HC microscope (Leica Microsystems, Wetzlar, Germany) with a Digital Sight DS-Fi1 and the DS-L2 control unit (Nikon, Tokyo, Japan).

### Production and purification of recombinant proteins

SOLD1 recombinant protein was produced by two different methods. Large amounts of protein were produced for antibody production using an automated, cell-free rapid translation system (RTS). However, the glycosylated protein could not be produced by RTS, and was generated using a HEK 293 mammalian cell expression system. The specificity of the custom-made antibody for the glycosylated protein was confirmed by western blot analysis.

#### Recombinant protein production by RTS

Bovine cDNA encoding mature SOLD1 protein regions was cloned by RT-PCR. The amino terminal of the mature protein was identified based on the consensus motif R-X-X-R, which is the targeting sequence of subtilisin-like proteases. Cloned sequences were subcloned into the pIVEX 2.4d expression vector (Roche), and used for cell-free protein expression. The protein was expressed using bacterial lysate reagent (RTS Proteomaster HY Kit, Roche), according to the manufacturer's instructions. The RTS reaction chamber used in the present study contained 1 ml of reaction mixture and 11 ml of feeding solution. After 24 h of incubation, 1 ml of reaction mixture was harvested, solubilized with 4 ml of 8 M urea solution, and centrifuged at 22000 g for 10 min at 4°C to collect the supernatant. Recombinant proteins were purified from the supernatant by chromatography on a Ni Sepharose 6 Fast Flow column (GE Healthcare, Buckinghamshire, UK) in the presence of 6 M urea.

#### Recombinant protein production by HEK 293 cells

The SOLD1 sequences encoding the mature protein region, which included the FLAG and 6x His epitope tag sequences, were inserted into a pFLAG-CMV-3 vector (Sigma). The constructed plasmid was transiently transfected into HEK 293 cells using FuGENE 6 (Roche Diagnostics, Basel, Switzerland). Stably transfected HEK 293 cells were adapted to suspension culture in a spinner flask using 293 SFM II medium (Invitrogen, Gibco), and cultured in an atmosphere of 5% CO_2_ in air at 37°C for three days. The medium was separated by centrifugation.

Recombinant FLAG-tag and 6x His-tag fusion proteins were purified using the 6x His-tag portion. Approximately 1 l of conditioned medium was processed at a time. Medium to which 1 ml Ni Sepharose 6 Fast Flow (Amersham Bioscience, Buckinghamshire, UK) was added was mixed and equilibrated with 20 mM sodium phosphate buffer, pH 8.0, containing 300 mM NaCl and 20 mM imidazole. Only the 6x His-tag proteins bind to the Ni Sepharose 6 Fast Flow carrier. The medium with carrier was chromatographed on a PD-10 column (Amersham Bioscience). The fractions with carrier were washed with 20 mM imidazole. The fractions were eluted with 250 mM imidazole.

### Anti-bovine SOLD1 antibody production

Anti-bSOLD1 antibody was generated in rabbits. The rabbits were bled prior to the immunization to obtain preimmune serum. They were initially inoculated with 300 µg of the antigen in Freund's complete adjuvant. Three weeks after the initial immunization, the rabbits were further inoculated with 150 µg of the antigen in Freund's incomplete adjuvant. Animals were given three booster injections at two-week intervals. The titer of antiserum was monitored by ELISA. Two weeks after the third booster injection, the animals were exsanguinated to collect antiserum.

### Western blot analysis

One nanogram of purified SOLD1 recombinant protein was loaded on each lane, separated by SDS-PAGE, and electrophoretically transferred onto a polyvinylidene-difluoride membrane. The membrane was blocked in 10% skimmed milk overnight and incubated with custom-made bovine anti-bSOLD1 antibody for 1 h at room temperature, followed by incubation with anti-rabbit IgG conjugated with alkaline phosphatase (Sigma) (diluted 1∶3000) for 1 h at room temperature. Immunopositive bands were stained using NBT (Bio-Rad, Hercules, CA, USA) and BCIP (Bio-Rad).

### Immunohistochemistry

Immunohistochemistry was also performed using the automated Ventana HX System Discovery with the reagents DabMapKit (Ventana). The 7 µm-thick sections were incubated with anti-bSOLD1 antibody, anti-collagen antibody of type-I, or anti-collagen antibody of type-III (CosmoBio, Tokyo Japan), at a dilution of 1∶100 (anti-bSOLD1) or 1∶20 (anti-collagen) in Ab Diluent (Ventana) for 4 h. The sections were then washed and incubated with anti-rabbit IgG-Biotin conjugate (Sigma) for 1 h. Immunoreactive signals were detected using streptavidin-HRP and diaminobenzidine (DabMapKit, Ventana). Counter stain was performed by hematoxylin and bluing reagent (saturated lithium carbonate solution). After treatment, the sections were observed with a Nikon ECLIPSE E800 photomicroscope equipped with a DS-Fi1 Digital Camera and a DS-L2 control unit (Nikon, Tokyo, Japan).

### Extracellular matrix (ECM)-coated microplate binding assay

We prepared four types of commercial ECM-coated 96-well microplates (COL1, type IV collagen (COL4), fibronectin (FN), and laminin (LAM)), and a poly-L-lysine (negative control) 96-well microplate (BD Biosciences, San Jose, CA, USA). These were used to assess SOLD1 protein interactions with each component of the ECM. In addition, the 96-well microplates that were manually coated with COL1 (telopeptide including COL1-A or excluding COL1-P), COL4 and COL3 (300 µg/well each) (Cellmatrix type I-A, I-P, IV or III collagens; Nitta-gelatin, Tokyo, Japan) were used to investigate the concentration-dependent binding of immobilized collagens to the SOLD1 protein (60 ng/well). For the binding assay, the microplates were blocked with 5% non-fat dry milk in TBS for 1 h at room temperature (RT), and washed three times with TBS containing 0.1% Tween 20 (TBST). Then, 100 µl of 1% nonfat dry milk in TBS containing recombinant SOLD1 protein produced by the mammalian cell system (dilution 1∶300; 60 ng/well) were dispensed into microplates, incubated for 1 h at RT, and then washed three times with TBST. Further, 100 µl of 1% nonfat dry milk in TBS containing antiserum (dilution 1∶500) were dispensed into microplates and incubated for 1 h at RT and then washed three times with TBST, and 100 µl of 1% nonfat dry milk in TBS with anti-rabbit IgG alkaline phosphatase conjugate (Sigma) (dilution 1∶1000) were dispensed into microplates and incubated for 1 h at RT, and then washed three times with TBST. The ECM-bound SOLD1 was colored by p-nitrophenylphosphate (Sigma). The colorimetric analysis was performed by monitoring absorbance at 405 nm using the 1420 Muliabel Counter ARVO MX (Perkin Elmer, Waltham, MA, USA).

### Detection of polarized secretion of SOLD1 in a bovine trophoblast cell line (BT-1)

BT-1 is a bovine trophoblast cell line that was established in our laboratory [64], [65]. The apico-basal polarity of SOLD1 secretion was examined in the BT-1 cells, after confirming *SOLD1* expression by RT-PCR. BT-1 cells have apico-basal polarized properties. The apical face of the cell contacts the culture medium, and the basolateral face contacts the collagen-coated plate. A secreted protein is present in the conditioned medium when it is secreted from the apical face, and adheres to the collagen-coated plate when it is secreted from the basolateral face. A 96-well microplate manually coated with COL1-A (300 µg/well) (Nitta-gelatin) was used for BT-1 culture. BT-1 was cultured in Dulbecco's modified Eagle's/F-12 medium (DME/F-12, Sigma) containing 100 IU/ml of penicillin and 100 µg/ml of streptomycin (Sigma), supplemented with 20% fetal bovine serum (FBS) at 37°C in an atmosphere of 5% CO_2_. Western blotting of the conditioned medium was performed to detect secretion of SOLD1 from the apical face, and the microplate binding assay was performed to detect secretion from the basal face of the cells, after the cells were removed and the microplate was washed with ten times with TBST by Wallac 1296-026 Delfia Platewasher (Perkin Elmer). The cells are completely torn off from the plate by the hydraulic pressure of the plate washer, because the adhesion of BT-1 cells to the plate is weak. Expression of SOLD1 by BT-1 cells was confirmed by western blotting of the cell lysate.

### Multiple alignment of the deduced protein sequences

The deduced SOLD1 protein sequences were aligned with members of the Ly-6 superfamily from various species with the multiple alignment software Clustal W 1.83 on the DDBJ website [http://clustalw.ddbj.nig.ac.jp/top-j.html], using the Neighbor-Joining (NJ) method [66]. Domain retrieval of the SOLD1 protein was performed using the ProDom website [http://prodomweb.univ-lyon1.fr/prodom/current/html/home.php].

### Genomic organization of *SOLD1*

The genomic organization of *SOLD1* was investigated using the database in the NCBI Map Viewer web site [http://www.ncbi.nlm.nih.gov/mapview/].
